# Supplementary material for: Pool size estimations for dense‐core vesicles in mammalian CNS neurons
Source: EMBO J. 2018 Sep 5;37(20):e99672. doi: 10.15252/embj.201899672 (PMC6187028; doi:10.15252/embj.201899672)
Supplement: Supplementary file 1 — Expanded View Figures PDF [file EMBJ-37-e99672-s001.pdf]

Expanded View Figures

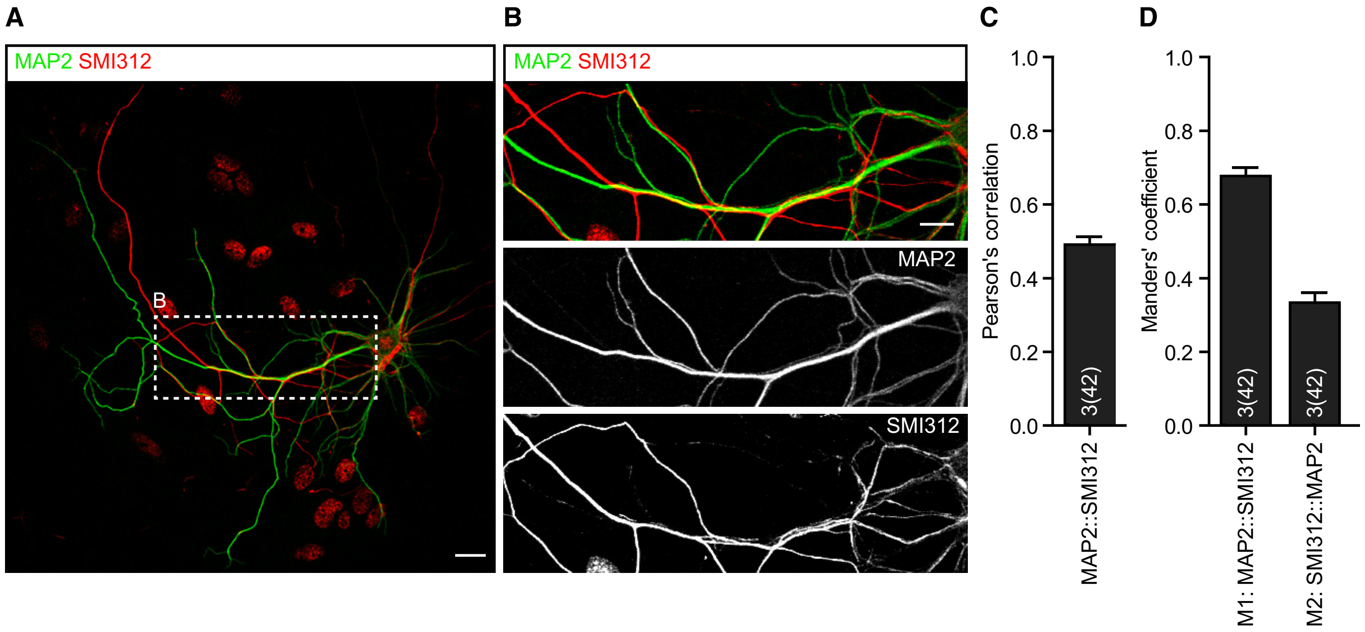

Figure EV1. Axons and dendrites significantly overlap in single isolated hippocampal neurons.

A Representative confocal image of a single isolated hippocampal neuron (DIV14) immunostained for the dendritic marker MAP2 (green) and the axonal marker SMI312 (red). Scale bar: 20 μm.

B Zoom of area indicated in (A) showing axons frequently running parallel to dendrites. Scale bar: 10 μm.

C Pearson's correlation of colocalization analysis between MAP2 and SMI312.

D Manders' coefficients of colocalization analysis between MAP2 and SMI312.

Data information: Bars show mean ± SEM. *N* numbers represent number of experiments and number of individual observations (neurons) in brackets.

© 2018 The Authors

© 2018 The Authors *The EMBO Journal* e99672 | 2018 19 of 18

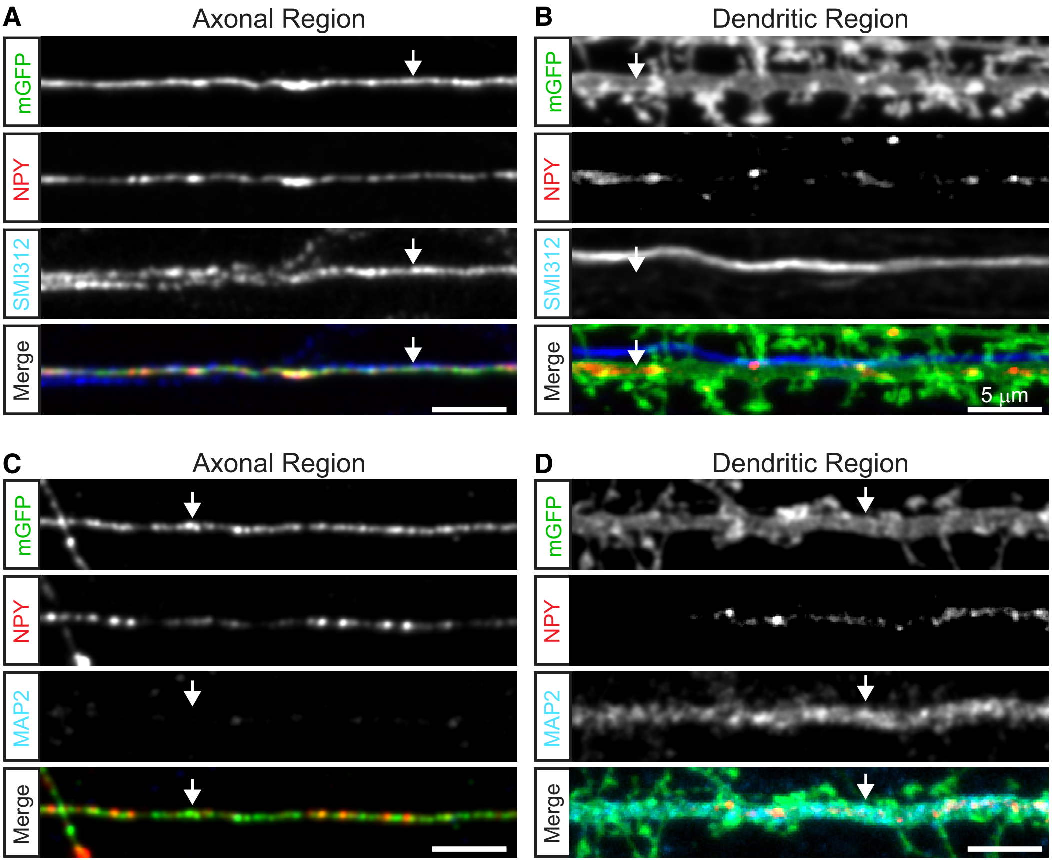

Figure EV2. Identification of axons and dendrites in sparse-labeled high-density culture.

A–D Representative confocal zooms of high-density hippocampal neuron culture (DIV14) sparse-labeled with membrane-targeted EGFP (mEGFP) and NPY-mCherry, immunostained for the axonal marker SMI312 (A, B) or dendritic marker MAP2 (C, D). Scale bars: 5 μm. (A) Zoom of axon labeled with membrane-targeted EGFP and positive for the axonal maker SMI312 (indicated by arrows). (B) Zoom of dendrite labeled with membrane-targeted EGFP and negative for the axonal marker SMI312 (indicated by arrows). (C) Zoom of axon labeled with membrane-targeted EGFP and negative for the dendrite marker MAP2 (indicated by arrows). (D) Zoom of dendrite labeled with membrane-targeted EGFP and positive for the dendrite marker MAP2 (indicated by arrows).

© 2018 The Authors

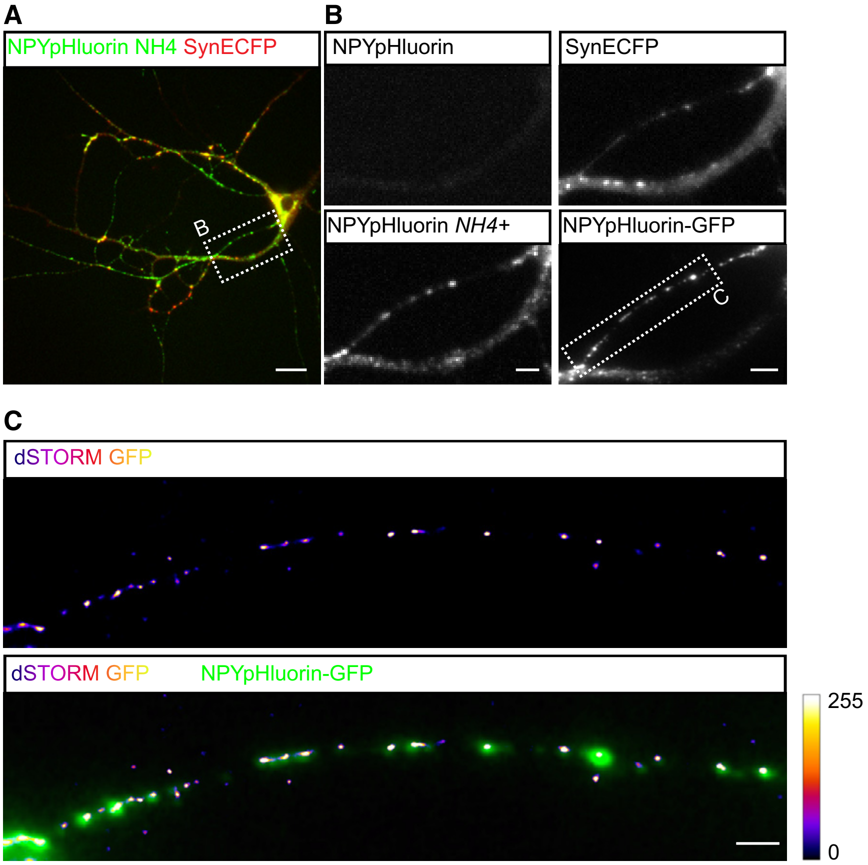

Figure EV3. ***d*STORM imaging of NPY-pHluorin.**

A Still of live hippocampal neuron (DIV 14) infected with NPY-pHluorin (green) and synapse marker Syn-ECFP (red), superfused with NH_4-Tyrodes to visualize the total NPY-pHluorin pool. Scale bar: 20 µm.

B Zoom of region indicated in (A). Live NPY-pHluorin signal before NH_4-Tyrodes application (upper left). Live NPY-pHluorin signal upon NH_4-Tyrodes application (lower left). Live Syn-ECFP signal (upper right). *Post hoc* immunoreactivity of NPY-pHluorin labeled with anti-GFP (lower right). Scale bars: 5 µm.

C Reconstructed GFP localizations of *d*STORM imaging of zoomed region indicated in (B) (lower right). Lower panel shows overlay between *d*STORM GFP localizations and NPY-pHluorin-GFP immunoreactivity (confocal, 40×). Calibration bar represents no (0) to high (255) clustering of *d*STORM localizations. Scale bars: 2 µm.

© 2018 The Authors
